# Self-perceived competence and training needs analysis on antimicrobial stewardship among government ward pharmacists in Malaysia

**DOI:** 10.1093/jacamr/dlaa035

**Published:** 2020-07-16

**Authors:** Cherh Yun Teoh, Adliah Mhd. Ali, Noraida Mohamed Shah, Rohana Hassan, Chee Lan Lau

**Affiliations:** 1 Centre of Quality Management of Medicines, Faculty of Pharmacy, Universiti Kebangsaan Malaysia, Kuala Lumpur Campus, Kuala Lumpur, Malaysia; 2 Pharmacy Department, Hospital Kuala Lumpur, Kuala Lumpur, Malaysia; 3 Pharmacy Department, Hospital Canselor Tuanku Muhriz, Universiti Kebangsaan Malaysia, Kuala Lumpur, Malaysia

## Abstract

**Background:**

There is a paucity of data on pharmacists’ competency and learning needs in antimicrobial stewardship (AMS).

**Objectives:**

To identify and prioritize learning needs based on self-perceived competence of ward pharmacists in AMS, to identify predictors of self-perceived competence, learning methods in AMS and perceived barriers to learning.

**Methods:**

A cross-sectional survey involving ward pharmacists from Hospital Canselor Tuanku Muhriz (HCTM) and hospitals under the Ministry of Health was conducted from May to July 2018.

**Results:**

A total of 553 ward pharmacists from 67 hospitals responded to this survey (71.3% response rate). Knowledge of infections, antimicrobials and AMS systems, confidence to advise on various issues relating to antimicrobial therapy and participation in clinical audit and evaluation were among the learning needs identified (median score 3.00). Meanwhile, knowledge on the epidemiology of infections, off-label use of antimicrobials and pharmacoeconomics relating to antimicrobials had lower median scores (2.00) and were thus prioritized as high learning needs. Significant predictors of self-perceived competence in AMS were: gender (*P *<* *0.001); prior specific training in infections and AMS (*P *<* *0.001); postgraduate degree (*P *<* *0.001); practising in the area of infectious disease (*P *<* *0.05); and years of working experience as a ward pharmacist (*P *<* *0.005). Continuing medical education, seminars, courses and workshops were the most common (78.1%) and preferred (84.6%) learning methods in AMS. Lack of appropriate training (67.8%), time (44.5%) and funding (42.5%) topped the list of barriers to learning in AMS.

**Conclusions:**

Findings in this study suggest the need to establish and intensify standardized training in AMS among government ward pharmacists.

## Introduction

Antimicrobial resistance is a major threat to global health, estimated to account for 10 million deaths annually by the year 2050.^1^ To curb this issue, the concept of antimicrobial stewardship (AMS) was first suggested by McGowan and Gerding^2^ to highlight that antimicrobials should only be used with care when indicated.

AMS is defined by IDSA as a set of coordinated interventions designed to improve and measure the appropriate use of antimicrobial agents by promoting the selection of optimal antimicrobial drug regimen, dose, duration of therapy and route of administration.^3^ The impact of AMS programmes on antimicrobial resistance is well documented in past literature, with evidence showing a pooled reduction in total antimicrobial consumption, a decrease in the use of restricted antimicrobial agents^4^ and a substantial reduction of the relative risk of mortality.^5^

Among the stakeholders in AMS, pharmacists played and continue to play a key role. Throughout the development of AMS in the 1990s and 2000s, pharmacists spearheaded AMS teams or took pivotal roles as co-leaders, together with infectious disease specialists or clinical microbiologists.^6^ The CDC has defined pharmacist-driven roles and interventions to include IV-to-oral switch for antimicrobials, therapeutic monitoring with dose adjustments and optimization when necessary, alert for duplicative or unnecessary antimicrobial therapy, stop orders for antimicrobials intended only for short duration (e.g. surgical prophylaxis) and detection of antibiotic-related drug–drug interactions.^7^

The CDC has also acknowledged the critical role of pharmacists in prospective audit of antimicrobial therapy, disease state management for infections, review of clinical information for antibiotic timeouts and provision of direct feedback to prescribers on AMS-related issues.^7^ The involvement of pharmacists in AMS was shown to contribute to improved timeliness of the administration for in-ward antimicrobial agents,^8^ shortened hospital stay,^9^ significant reduction of antibiotic consumption^10^ and a lower rate of hospital mortality and MDR.^11^ It is believed that these successful outcomes of AMS are much attributable to the pharmacists’ competence based on their knowledge and clinical skills obtained through training and experience.^12^

In Malaysia, the Ministry of Health launched AMS for government healthcare facilities in 2014. While the roles of pharmacists specified in local guidelines for AMS are similar to the pharmacist-driven interventions by the CDC, the Malaysian protocol has several extra roles outlined. These include technical guidance regarding antimicrobials and newer agents, restriction of specific antimicrobials (which usually pose a high risk of antimicrobial resistance), collaboration with and education of ward pharmacists to identify potential patients for AMS interventions and the surveillance of antimicrobial usage.^13^

However, since the implementation of AMS in government healthcare facilities in Malaysia, no standardized training modules for pharmacists in infectious disease and AMS have been available. Hence, this study serves as a preliminary initiative to identify the level of self-perceived competence and learning needs, predictors of self-perceived competence, currently available and preferred learning methods and barriers to learning in AMS among ward pharmacists in Malaysia.

## Methods

This was a cross-sectional descriptive survey involving ward pharmacists working in hospitals under the Ministry of Health and the Ministry of Higher Education in Malaysia, conducted from May to July 2018. Ward pharmacists and other pharmacists who are involved in the activities of ward pharmacy for at least 10 h per week were included.

Convenience sampling was applied in this study. The sample size was calculated based on the equation by Li^14^ for descriptive surveys, which yielded a minimum number of 384. However, since the total population of government ward pharmacists in Malaysia is less than 10 000, the final estimated sample size was at least 200 respondents.

The data collection tool contained sections on demographic characteristics, self-perceived competence in AMS, current and preferred learning methods in AMS and barriers to learning in AMS.

A total of 28 questions with 5-point Likert Scale options were used to assess self-perceived competence in AMS (available as Supplementary data at *JAC-AMR* Online). These questions were adapted and modified from the recommended lists of competencies in the Royal Pharmaceutical Society’s Expert Professional Practice Curriculum for Pharmacists in Infections and Antimicrobial Stewardship.^15^ Questions were categorized into four constructs: six questions on the level of knowledge; eight questions on the level of confidence; three questions on attitudes; and three questions on the frequency of practice. Different types of Likert scale option anchors were chosen based on the constructs mentioned.^16^ Each of the Likert scale responses carried a score, which was calculated in total to reflect the overall self-perceived competence of government ward pharmacists in this study; higher total scores indicated higher levels of self-perceived competence in AMS.

The subsequent section of the questionnaire consisted of items pertaining to current and preferred learning methods. Two multiple-choice questions with the same lists of learning methods were provided by adopting the options in a learning-needs analysis of pharmacists.^17^

The final section of the questionnaire dealt with barriers to learning in AMS. The options were adapted from another learning-needs analysis of clinical pharmacists and included constraints related to lack of employer support, funding, time, appropriate training on offer, motivation and uncertainty regarding the quality of available training.^18^

The first draft of this questionnaire was sent for expert review to one AMS pharmacist and one ICU pharmacist in a tertiary hospital to establish face and content validity. Amendments and suggestions, including restructuring and removal of questions, were made to produce a pre-piloted questionnaire. Subsequently, a pilot test was conducted and Cronbach’s α values were measured from the results of the pilot test to determine the internal consistency of the questions. Further amendments involving the removal or substitution of questions were done based on the findings of the pilot studies. Three different rounds of pilot studies were conducted from March to April 2018. A total of 30 government ward pharmacists were involved in these pilot studies and were excluded from the actual study. The finalized questionnaire achieved an overall Cronbach’s α value of 0.9, which is above the acceptable level shown in literature,^19^ and was attributed to higher levels of correlation between the questions studied.

The finalized questionnaire was produced in both hard copy and the form of electronic survey via Google Forms. Each hospital was approached individually to obtain institutional approval from both the director and the chief pharmacist. Upon receipt of approval, further phone or electronic communication facilitated identification of pharmacy department representatives, access to a directory of ward pharmacists and their corresponding email addresses and feedback on preferred mode of completing the questionnaire (i.e. either via the online platform or by the use of a hard copy). The URL of the electronic survey was emailed to respective ward pharmacists or pharmacist representatives of the participating hospitals for internal dissemination among ward pharmacists under their care. Printed questionnaires were dispatched to ward pharmacists who opted to answer on a hard copy. Each of the questionnaires was coded with a different serial number to avoid confusion and to facilitate data retrieval when necessary. Reminder emails were sent to the ward pharmacists to improve the response rate.

IBM SPSS Statistics Version 23.0 was used to analyse the study findings. Descriptive statistics were used to describe demographic data of the respondents and distributions of response for other questions listed in the questionnaire. For the evaluation of individual items or constructs of self-perceived competence based on the Likert scales, a median score of 3 is considered a priority in learning need, while a score of less than 3 is deemed high priority in learning need.^18^ The *χ*^2^ test was used to identify any association between demographic characteristics of the respondents and their status of prior specified training in infections and AMS, while blockwise multiple linear regression was used to identify predictors affecting the levels of self-perceived competence among the respondents.

This study was registered with the National Medical Research Registry (NMRR-17-3194-39210). Ethical approval was obtained from the Medical Research Ethics Committee (MREC) in the Ministry of Health, Malaysia [KKM.NIHSEC.P19-394(5) and KKM/NIHSEC/P19-394(7)] and the Research Ethics Committee of Universiti Kebangsaan Malaysia (UKM PPI/111/8/JEP-2018-120) before commencement of the study.

## Results

A total of 553 ward pharmacists from 67 hospitals across the nation, including 66 Ministry of Health hospitals and Hospital Canselor Tuanku Muhriz (a university hospital) participated in this study. A response rate of 71.3% was achieved, with a total inclusive population of 776 eligible government ward pharmacists in all participating hospitals. Three hundred and ninety-one respondents (70.7%) provided their feedback via electronic survey while the remaining 162 responses (29.3%) were received via hard copy.

### Demographic characteristics

The demographic characteristics of the respondents are illustrated in Table 1. The mean age of the respondents was 31.04 ± 3.50 years, while the average duration of working experience as a ward pharmacist was 4.27 ± 3.30 years. The majority of the respondents were female (*n *=* *464, 83.9%), aged 30–39 years (*n *=* *335, 60.6%), had a Bachelor’s degree as their current highest academic qualification (*n *=* *428, 77.4%), did not receive prior specific training in infections and AMS (*n *=* *421, 76.1%) and only had 1–5 years of working experience as a ward pharmacist (*n *=* *332, 60.0%). There were 12 (2.2%) respondents who practised in the area of infectious disease. However, only five of them (41.7%) received prior specific training in infections and AMS. *χ*^2^ tests between prior training and other demographic characteristics did not demonstrate statistical significance, except for age and duration of working experience as a ward pharmacist.

**Table 1.** Demographic characteristics of the respondents (*N *=* *553)

Characteristics	Received prior specific training on infections and AMS (*n *=* *132)		Did not receive prior specific training on infections and AMS (*n *=* *421)		*P* value (*χ*^2^)
	frequency, *n*	%	frequency, *n*	%	
Gender					
male	22	4.0	67	12.1	0.837 (0.042)
female	110	19.9	354	64.0	
Age (years)					
20–29	37	6.7	171	30.9	<0.001 (29.897)
30–39	86	15.6	249	45.0	
≥40	9	1.6	1	0.2	
Highest academic qualification					
Bachelor’s degree	95	17.2	333	60.2	0.088 (2.918)
Master’s degree and above	37	6.7	88	15.9	
Hospital settings					
main state hospital, university hospital and special medical institution	67	12.1	197	35.6	0.752 (1.203)
major specialist hospital	45	8.1	161	29.1	
minor specialist hospital	19	3.4	57	10.3	
non-specialist hospital	1	0.2	6	1.1	
Discipline of practice					
general medical	45	8.1	164	29.7	0.241 (2.846)
infectious disease	5	0.9	7	1.3	
others	82	14.8	250	45.2	
Duration as a ward pharmacist (years)					
<1	3	0.5	44	8.0	<0.001 (34.175)
1–5	63	11.4	269	48.6	
6–10	54	9.8	97	17.5	
>10	12	2.2	11	2.0	

### Self-perceived competence and learning needs in AMS

Table 2 lists the responses provided pertaining to questions about self-perceived competence in AMS. Total scores of self-perceived competence achieved in this study ranged between 45 and 137 (mean score 84.33 and median score 84.00). All items about knowledge assessment were identified as learning needs (median scores 3.00), including the knowledge of epidemiology in infection and off-label use of antimicrobials, which were identified as high learning needs (median scores 2.00). For the assessment of confidence, all items were identified as learning needs (median scores 3.00), including confidence in providing advice on pharmacoeconomic issues relating to antimicrobials identified as high priority in learning needs (median scores 2.00). None of the items under the section of self-perceived attitude was identified as a learning need (median scores 4.00). The only learning needs identified in the section on the self-perceived frequency of practice were participation in clinical audit and evaluation relating to antimicrobial pharmacy service (median score 3.00).

**Table 2.** Summary of self-perceived competence in AMS among government ward pharmacists in Malaysia (*N* = 553)

Variables	Number of respondents, *n* (%)					Mean score* *±* *SD	Median score
	I know nothing	I know a little	I know an adequate amount	I know a lot	I am an expert		
All types of infections and comorbidities including:							
aetiology	12 (2.2)	216 (39.1)	302 (54.6)	21 (3.8)	2 (0.4)	2.61* *±* *0.616	3.00
physiology	7 (1.3)	247 (44.7)	272 (49.2)	24 (4.3)	3 (0.5)	2.58* *±* *0.623	3.00
common signs and symptoms	2 (0.4)	156 (28.2)	335 (60.6)	55 (9.9)	5 (0.9)	2.82* *±* *0.635	3.00
epidemiology	33 (6.0)	275 (49.7)	230 (41.6)	13 (2.4)	2 (0.4)	2.41* *±* *0.657	2.00
risk factors	5 (0.9)	182 (32.9)	316 (57.1)	46 (8.3)	4 (0.7)	2.75* *±* *0.645	3.00
Details of antimicrobials including:							
mechanism of action	1 (0.2)	135 (24.4)	357 (64.6)	54 (9.8)	6 (1.1)	2.87* *±* *0.614	3.00
indications	1 (0.2)	61 (11.0)	367 (66.4)	115 (20.8)	9 (1.6)	3.13* *±* *0.613	3.00
adverse effects and precautions	1 (0.2)	110 (19.9)	367 (66.4)	71 (12.8)	4 (0.7)	2.94* *±* *0.600	3.00
drug interactions	5 (0.9)	177 (32.0)	321 (58.0)	47 (8.5)	3 (0.5)	2.76* *±* *0.636	3.00
off-label use of drugs	23 (4.2)	299 (54.1)	202 (36.5)	27 (4.9)	2 (0.4)	2.43* *±* *0.670	2.00
Therapeutic management of patients with infections	2 (0.4)	87 (15.7)	379 (68.5)	80 (14.5)	5 (0.9)	3.00* *±* *0.594	3.00
Policies, procedures and treatment guidelines	3 (0.5)	117 (21.2)	358 (64.7)	69 (12.5)	6 (1.1)	2.92* *±* *0.630	3.00
Interpretation of lab tests and/or disease markers	3 (0.5)	102 (18.4)	362 (65.5)	81 (14.6)	5 (0.9)	2.97* *±* *0.623	3.00
System of the antimicrobial stewardship service	14 (2.5)	156 (28.2)	284 (51.4)	94 (17.0)	5 (0.9)	2.86* *±* *0.755	3.00
	Not confident at all	Not very confident	Somewhat confident	Very confident	Extremely confident		
Identify and manage patients with complex pharmaceutical care issues	6 (1.1)	124 (22.4)	341 (61.7)	77 (13.9)	5 (0.9)	2.91* *±* *0.660	3.00
Recommend appropriate monitoring parameters	2 (0.4)	69 (12.5)	357 (64.6)	118 (21.3)	7 (1.3)	3.11* *±* *0.627	3.00
Advise on pharmacokinetic and pharmacodynamic principles of antimicrobials	11 (2.0)	139 (25.1)	300 (54.2)	92 (16.6)	11 (2.0)	2.92* *±* *0.755	3.00
Advise on antimicrobial optimization	4 (0.7)	104 (18.8)	309 (55.9)	126 (22.8)	10 (1.8)	3.06* *±* *0.717	3.00
Advise on relevant policies and procedures in antimicrobial stewardship	18 (3.3)	170 (30.7)	275 (49.7)	81 (14.6)	9 (1.6)	2.81* *±* *0.783	3.00
Advise on relevant pharmacoeconomic issues relating to antimicrobials	46 (8.3)	235 (42.5)	222 (40.1)	45 (8.1)	5 (0.9)	2.51* *±* *0.797	2.00
Support other staff in aspects of pharmaceutical and related care of patients with infections	6 (1.1)	102 (18.4)	321 (58.0)	113 (20.4)	11 (2.0)	3.04* *±* *0.715	3.00
Make an informed decision timely, based on analysed evidence, with the ability to defend your decisions accordingly	5 (0.9)	125 (22.6)	330 (59.7)	85 (15.4)	8 (1.4)	2.94* *±* *0.686	3.00
	Strongly disagree	Disagree	Neither agree or disagree	Agree	Strongly agree		
I am able to consider/take into account various factors and situations while making clinical judgement pertaining to antimicrobial pharmacotherapy	3 (0.5)	10 (1.8)	123 (22.2)	388 (70.2)	29 (5.2)	3.78* *±* *0.589	4.00
I am not able to work in the absence of senior’s support	77 (13.9)	277 (50.1)	140 (25.3)	55 (9.9)	4 (0.7)	3.67* *±* *0.863	4.00
I am not able to recognize my own limitations and refer appropriately to others within and outside own team	90 (16.3)	295 (53.3)	116 (21.0)	48 (8.7)	4 (0.7)	3.76* *±* *0.853	4.00
	Never	Rarely	Sometimes	Often	Always		
Apply organization policies, procedures and guidance related to local and national antimicrobial pharmacy service	28 (5.1)	36 (6.5)	156 (28.2)	230 (41.6)	103 (18.6)	3.62* *±* *1.022	4.00
Initiate or participate in planning or implementation of clinical audit and evaluation of the antimicrobial pharmacy service	92 (16.6)	127 (23.0)	176 (31.8)	110 (19.9)	48 (8.7)	2.81* *±* *1.186	3.00
Provide advice to other healthcare professionals on various situations pertaining to antimicrobial use	9 (1.6)	46 (8.3)	193 (34.9)	214 (38.7)	91 (16.5)	3.60* *±* *0.914	4.00

### Predictors of self-perceived competence in AMS

Findings of the multiple linear regression analysis are portrayed in Table 3. Five specific significant predictors were identified: gender (*P *<* *0.001); highest academic qualification of Master’s degree and above (*P *<* *0.001); area of practice in infectious disease (*P *<* *0.05); prior specific training in infections and AMS (*P *<* *0.001); and duration of working experience as a ward pharmacist (*P *<* *0.005). Among these predictors, prior specific training in infections and AMS ranked as the most significant predictor based on the corresponding β standardized coefficient value of 0.193. The *R*^2^ value of 0.194 implies that the significant predictors in this regression analysis altogether accounted for 19.4% influence towards the total scores of self-perceived competence in this study.

**Table 3.** Multiple linear regression analysis to identify predictors of self-perceived competence in AMS among government ward pharmacists in Malaysia (*N *=* *553)

Variable	β unstandardized coefficients	Standard error	β standardized coefficients	*t* value	*P* value	95% CI for β		*R* ^2^	*F*
						lower bound	upper bound		
Constant	86.152	1.720		50.080	<0.001	82.773	89.531	0.194	14.487 (*P *<* *0.001)
Female gender	−6.786	1.376	−0.190	−4.932	<0.001	−9.488	−4.083		
Master’s degree and above	5.718	1.311	0.183	4.360	<0.001	3.142	8.294		
Major specialist hospitals	0.172	1.109	0.006	0.155	0.877	−2.007	2.352		
Minor specialist hospitals	−2.844	1.613	−0.075	−1.763	0.078	−6.013	0.324		
Non-specialist hospitals	−5.509	4.619	−0.047	−1.193	0.233	−14.582	3.563		
Discipline of practice in infectious disease	7.416	3.608	0.083	2.055	<0.05	0.328	14.504		
Other discipline of practice	−1.712	1.092	−0.064	−1.567	0.118	−3.858	0.434		
Prior specific training in infections and AMS	5.931	1.220	0.193	4.863	<0.001	3.535	8.328		
Duration of working experience as a ward pharmacist	0.567	0.176	0.143	3.226	<0.005	0.222	0.913		

### Current and preferred learning methods in AMS

Continuing medical education (CME), seminars, courses or workshops were the most popular learning methods available across various hospital settings (*n *=* *432, 78.1%) (Table S1). This was followed by peer discussions (*n *=* *339, 61.3%) and self-learning or reading (*n *=* *337, 60.9%). One hundred and eighty-four (33.3%) respondents were involved in journal clubs or study groups, while 166 (30.0%) respondents relied on experiential learning such as specialized AMS ward rounds. Project work or research had the least participation; only 82 respondents (14.8%) chose this option. Thirty-three respondents (6.0%) did not specify the currently available learning methods in their facilities.

Most preferred learning methods identified were similar to the frequency distributions of the currently available learning methods. The majority of respondents (*n *=* *468, 84.6%) still preferred to learn about AMS via CME sessions, seminars, courses or workshops. Peer discussions (*n *=* *328, 59.3%) and self-learning/reading (*n *=* *290, 52.4%) ranked second and third, respectively, in order of preference. Experiential or hands-on learning, which included attachment, ranked fourth (52.3%, *n *=* *289) among the respondents, followed by mentoring activities, including learning from visiting infectious disease physicians (*n *=* *242, 43.8%), and journal clubs or study groups (40.1%, *n *=* *222). Learning AMS via project work or research remained the least preferred method (*n *=* *84, 15.2%). Five respondents (0.9%) did not specify their preferred choice of learning methods in AMS.

### Barriers to learning in AMS

A few barriers to learning in AMS were identified among the respondents in this study (Table S2). More than half of the respondents acknowledged the lack of appropriate training as a major hindrance to learning in AMS. Lack of time to attend training (*n *=* *246, 44.5%) and the lack of funding (*n *=* *235, 42.5%) were the second and third highest ranked factors, respectively. One hundred and ninety-five (35.3%) respondents attributed barriers in learning to lack of organizational support while 121 (21.9%) expressed uncertainty regarding the quality of available training. The least acknowledged factor was lack of motivation to learn about AMS, which was recorded from 14.1% of the respondents (*n *=* *78).

Other barriers not listed as options included the multitasking role of ward pharmacists (i.e. other jobs not pertaining to ward pharmacy activities) (*n *=* *2, 0.4%), lack of case studies available (*n *=* *2, 0.4%), lack of self-discipline (*n *=* *1, 0.2%) and lack of AMS training providers in their hospitals (*n *=* *1, 0.2%). Only 1.8% of the respondents (*n *=* *10) did not specify any barriers to learning in AMS.

## Discussion

To the best of our knowledge, this is the first nationwide multicentre study to assess self-perceived competence on AMS among government ward pharmacists in Malaysia. In general, our participants had a moderate level of self-perceived competence in AMS. It suggests that fundamental knowledge on infections, antimicrobials, aspects of therapeutic management and the operation of AMS service need to be reiterated from time to time to sustain the competence of ward pharmacists in AMS. Findings of this study also call for action to strengthen the confidence of ward pharmacists in communicating issues relating to antimicrobials, especially in the aspect of pharmacoeconomics.

The relatively lower level of ward pharmacists’ involvement in clinical audit and evaluations on antimicrobial pharmacy service suggests that such activities were rarely performed in the respective facilities, or that they might be assigned to ward pharmacists in more advanced hierarchical positions. As audit and evaluation are key elements of AMS, with the potential to address knowledge gaps regarding infections and antimicrobial use among ward pharmacists, the findings of this study emphasize the need to integrate audit and evaluation in ward pharmacists’ roles.

The predictors of self-perceived competence in this study are well justified. Having prior specific training in infections and AMS as the strongest predictor suggests that specific training is always crucial to establish optimal levels of knowledge and confidence in performing AMS activities. The process of experiential learning during training often provides a holistic view of the essential requirements of skills and knowledge in AMS. Duration of working experience as a predictor was similarly reported in another study conducted among public health nurses in Japan.^20^ A longer duration of working experience provides more opportunities for pharmacists to encounter different types of cases and thereby strengthens their knowledge and skills from within, which reasonably explains its significance in this study. Practice in the area of infectious disease further consolidated pharmacists’ knowledge, hence the area of infectious disease was found to be a significant predictor of self-perceived competence in our study. Postgraduate studies in clinical pharmacy often require students to perform clerkship.^21^^,^^22^ This case-based experiential approach provides an opportunity for students to perform critical appraisals on the treatment regimen given and compare them with established clinical practice guidelines. Possibly, these learning steps helped to establish appropriate concepts on the management of infectious disease and hence may explain the higher level of confidence in this case.

The finding that female gender negatively correlates with self-perceived competence coincides with other input from currently available studies involving female participants in other career fields.^23^ Female participants not only tend to undervalue themselves but also have higher stress while self-evaluating.^24^ Studies also found a greater extent of negative recall biases in female participants compared with male participants, in which they have higher tendencies to recall their own mistakes and errors.^25^^,^^26^ As this may result in the tendency to underestimate their performance, it partially explains why female respondents scored lower in the assessment of self-perceived competencies in this study.

This study strongly indicates a high preference towards learning via CME sessions, seminars, courses and workshops among government ward pharmacists across the nation. This is consistent with the findings in another study involving Malaysian pharmacists in the government sector,^27^ as well as other studies involving pharmacists in the UK, Qatar, Egypt, Canada, the USA and Belgium.^17^^,^^28–32^ In fact, it further supports the notion that government sector pharmacists in Malaysia are still consistently inclined to traditional approaches of directed learning.^27^ The phenomenon of project work or research being the least preferred learning method is concurrent with other studies and could be attributed to pharmacists’ reluctance to participate in research activities.^17^^,^^27^^,^^33^ In fact, in two recent surveys pertaining to pharmacists’ participation in research, many confessed that they were incompetent in the aspects of research design and implementation.^33^^,^^34^ The lack of dedicated time and funding, increasing workload and lack of awareness of opportunities were also found to hinder pharmacists from participating in research.^33^

A higher proportion of respondents in this study preferred to learn about AMS via hands-on/experiential activities and mentoring activities, compared with the frequency of both activities chosen under current learning methods. It denotes an increased interest in and awareness of the importance of both mentoring activities and experiential learning.^17^ As implied from the significant improvement of students’ knowledge of and confidence in the skills of pharmacotherapy and patient care via human patient simulation, experiential learning allows tremendous growth of competency among pharmacists in AMS by providing direct experience.^35^ Mentoring activities help to nurture and instil appropriate values, knowledge, attitudes and perceptions in AMS via a close mentor–mentee relationship.

Assessment of barriers to learning implied that extrinsic factors were more predominant than intrinsic factors. However, the specific role of the paucity of appropriate training is considered a unique finding as it was not indicated in other similar studies involving pharmacists. In fact, current available literature commonly identified job constraints, lack of time and logistical factors such as location and distance of group learning activities as the significant barriers to continuing professional development and education among pharmacists.^27^^,^^28^

The development of AMS programmes in Malaysia is still considered to be in its infancy stage for many government healthcare facilities; hence the lack of availability of trainers or training activities is justified. It could also mean that there is a paucity of training activities that suit pharmacists with varying learning styles. This finding suggests that establishment of a standardized training framework and the Training of Trainers (ToT) programme for pharmacists in AMS is essential to meet the current demands among government ward pharmacists. It may also signify the necessity to identify various learning styles among pharmacists and tailor the approach of training in AMS accordingly in local settings.

This study managed to identify a number of learning needs, preferred learning methods and barriers to learning in AMS among government ward pharmacists in Malaysia. However, there was a paucity of respondents recruited from non-specialist hospitals under the Ministry of Health Malaysia, due to procedural constraints while obtaining institutional approval. At the same time, ward pharmacists from two other university hospitals were excluded due to issues pertaining to ethical approval application in both facilities. Some ward pharmacists from a few major specialist hospitals and non-specialist hospitals were recruited earlier for pilot studies. The levels of competence identified in this study may not reflect the actual competence, as they might be affected by both underestimation and overestimation of self-confidence while completing the questionnaire. Assessments utilizing observations and objective measurements, a higher representation of ward pharmacists from non-specialist hospitals and subgroup analysis of competence across different groups based on demographic characteristics of ward pharmacists should be considered in future studies.

### Conclusions

Government ward pharmacists in Malaysia were found to have a moderate level of self-perceived competence in AMS. Various aspects of knowledge, confidence and practice pertaining to AMS were identified as learning needs in this study. Prior specific training in infections and AMS most significantly predicted the total scores of self-perceived competence in AMS. The preferred learning methods were CME, seminars, courses or workshops; however, access to these are hampered by the lack of time and funding as well as a paucity of appropriate training available. Findings in this study highlight the need to establish and intensify standardized training to accommodate the increasing demands of learning in AMS among government ward pharmacists.

## Funding

This study was supported by Universiti Kebangsaan Malaysia (grant UKM GUP-2017-107).

## Transparency declarations

None to declare.

## Supplementary data

Tables S1 and S2, the Questionnaire and Reviewer reports 1 and 2 are available as Supplementary data at *JAC-AMR* Online.

## Supplementary Material

dlaa035_Supplementary_Data
