# Supplementary material for: Self-perceived competence and training needs analysis on antimicrobial stewardship among government ward pharmacists in Malaysia
Source: JAC Antimicrob Resist. 2020 Jul 16;2(3):dlaa035. doi: 10.1093/jacamr/dlaa035 (PMC8210314; doi:10.1093/jacamr/dlaa035)
Supplement: dlaa035_Supplementary_Data [file dlaa035_supplementary_data.zip › Supplementary_data.docx]

**Supplementary data**

Table S1. Distribution of current and preferred learning methods in antimicrobial stewardship among government ward pharmacists in Malaysia (N=553)

| **Learning Methods** | **Current** | | **Preferred** | | |
| --- | --- | --- | --- | --- | --- |
|  | **Frequency, n** | **%** | | **Frequency, n** | **%** |
| CME / seminar / course / workshop | 432 | 78.1 | | 468 | 84.6 |
| Self-learning / reading | 337 | 60.9 | | 290 | 52.4 |
| Journal clubs / study groups | 184 | 33.3 | | 222 | 40.1 |
| Peer discussion | 339 | 61.3 | | 328 | 59.3 |
| Experiential learning (hands-on) | 166 | 30.0 | | 289 | 52.3 |
| Mentoring | 75 | 13.6 | | 242 | 43.8 |
| Project work / research | 82 | 14.8 | | 84 | 15.2 |
| Did not specify | 33 | 6.0 | | 5 | 0.9 |

Table S2. Distribution of barriers to learning in antimicrobial stewardship among government ward pharmacists in Malaysia (n=553)

| **Barriers** | **Frequency** | |
| --- | --- | --- |
|  | **n** | **%** |
| Lack of appropriate training | 375 | 67.8 |
| Lack of time to attend training | 246 | 44.5 |
| Lack of funding | 235 | 42.5 |
| Lack of organizational support | 195 | 35.3 |
| Unsure of the quality of available training | 121 | 21.9 |
| Lack of motivation | 78 | 14.1 |
| Multitasking | 2 | 0.4 |
| Lack of case studies available for learning | 2 | 0.4 |
| Lack of self-discipline | 1 | 0.2 |
| Lack of training providers | 1 | 0.2 |
| Did not specify | 10 | 1.8 |

**A SURVEY ON SELF-PERCEIVED COMPETENCE AND LEARNING NEEDS ANALYSIS IN ANTIMICROBIAL STEWARDSHIP (AMS)
AMONG WARD PHARMACISTS IN MALAYSIA**

**_______________________________________________________________________________________**

**(A) DEMOGRAPHIC DATA**

| **Age :** ____________ years old | | **Gender :** Male / Female | |
| --- | --- | --- | --- |
| **Highest academic qualification :** □ Bachelor’s Degree □ Master Degree □ PhD | | | |
| **Duration of working as a ward pharmacist :** | | ______________ years | |
| **Current hospital setting ：** | | | |
| □ Main state hospital / university hospital | | | □ Special medical institution |
| □ Major specialist hospital | □ Minor specialist hospital | | □ Non-specialist hospital |
| **Discipline of practice :** | | | |
| □ Cardiology | □ Haematology | | □ Orthopaedic |
| □ Emergency Medicine | □ Infectious Disease | | □ Paediatrics |
| □ Endocrine | □ Intensive care | | □ Psychiatry |
| □ Gastroenterology | □ Nephrology | | □ Respiratory |
| □ General medical | □ Obstetric and Gynaecology | | □ Rheumatology |
| □ Geriatrics | □ Oncology | | □ Surgical |
| **Any prior specific training on infections and antimicrobial stewardship ?** □ Yes □ No | | | |
| **Duration of practice in the area of infectious disease: ____________** years | | | |

**(B) SELF-PERCEIVED COMPETENCE IN ANTIMICROBIAL STEWARDSHIP**

*Kindly place a* ***TICK (✓)*** *on the provided options* *based on your perceived ratings for each of the following questions.*

Questions no. 1 – 6 : Kindly rate your perceived level of knowledge on the elements stated below.

| No. | Knowledge | I know nothing | I know a little | I know an adequate amount | I know a lot | I am an expert |
| --- | --- | --- | --- | --- | --- | --- |
| 1 | All types of **infections and comorbidities** including : |  | | | | |
|  | (a) Aetiology |  |  |  |  |  |
|  | (b) Physiology |  |  |  |  |  |
|  | (c) Common signs and symptoms |  |  |  |  |  |
|  | (d) Epidemiology |  |  |  |  |  |
|  | (e) Risk factors |  |  |  |  |  |
| 2 | Details of **antimicrobials** including : |  | | | | |
|  | (a) Mechanism of action |  |  |  |  |  |
|  | (b) Indications |  |  |  |  |  |
|  | (c) Adverse effects and precautions |  |  |  |  |  |
|  | (d) Drug interactions |  |  |  |  |  |
|  | (e) Off-label use of drugs |  |  |  |  |  |
| 3 | **Therapeutic management** of patients with infections |  |  |  |  |  |
| 4 | **Policies, procedures and treatment guidelines** |  |  |  |  |  |
| 5 | **Interpretation** of **lab tests** and/or **disease markers** |  |  |  |  |  |
| 6 | **System** of the **antimicrobial stewardship service** |  |  |  |  |  |

Questions 7 – 14 : Kindly rate your perceived level of confidence on the abilities pertaining to pharmaceutical care needs in antimicrobial stewardship as stated below.

| No. | Skills | Not Confident at all | Not Very Confident | Somewhat Confident | Very Confident | Extremely Confident |
| --- | --- | --- | --- | --- | --- | --- |
| 7 | Identify and manage **patients with complex pharmaceutical care issues** |  |  |  |  |  |
| 8 | Recommend **appropriate monitoring parameters** |  |  |  |  |  |
| 9 | Advise on **pharmacokinetic and pharmacodynamics** principles of antimicrobials. |  |  |  |  |  |
| 10 | Advise on **antimicrobial optimization** |  |  |  |  |  |
| 11 | Advise on **relevant policies** and **procedures** in antimicrobial stewardship |  |  |  |  |  |
| 12 | Advises on **relevant pharmacoeconomic issues** relating to antimicrobials. |  |  |  |  |  |
| 13 | **Support other staff** in aspects of pharmaceutical and related care of patients with infections. |  |  |  |  |  |
| 14 | Make an **informed decision** timely based on analyzed evidence, with the ability to defend your decisions accordingly. |  |  |  |  |  |

Questions 15 - 17 : Kindly rate your level of agreement with the statements below.

| No. | Statement | Strongly disagree | Disagree | Neither agree or disagree | Agree | Strongly agree |
| --- | --- | --- | --- | --- | --- | --- |
| 15 | I am **able** to **consider /take into account various factors and situations** while making clinical judgement pertaining to antimicrobial pharmacotherapy. |  |  |  |  |  |
| 16 | I am **not able** to work in the **absence of senior’s support**. |  |  |  |  |  |
| 17 | I am **not able** to **recognize my own limitations** and **refer appropriately** to others within and outside own team. |  |  |  |  |  |

Questions 18 - 20 : Kindly rate your level of frequency in conducting the following practices in antimicrobial stewardship.

| No. | Practice | Always | Often | Sometimes | Rarely | Never |
| --- | --- | --- | --- | --- | --- | --- |
| 18 | **Apply** **organization policies, procedures** and **guidance** related to local and national antimicrobial pharmacy service. |  |  |  |  |  |
| 19 | **Initiate or participate** in planning or implementation of **clinical audit and evaluation** of the antimicrobial pharmacy services. |  |  |  |  |  |
| 20 | Provide **advice** **to other healthcare professionals** on various situations pertaining to antimicrobial use. |  |  |  |  |  |

**(C) CURRENT LEARNING METHODS IN ANTIMICROBIAL STEWARDSHIP**

*Kindly place a* ***TICK (✓)*** *at*  *the* *current educational activities pertaining to antimicrobial stewardship which are available at your facility. You may choose more than 1 option for this question or you may specify other options which are not listed here. You may also leave this section blank if there isn’t any educational activities being done at your facility pertaining to AMS.*

| □ CME / seminar / workshop /  course / conference | □ Self-learning / reading | □ Journal clubs / study groups |
| --- | --- | --- |
| □ Peer discussions | □ Experiential learning  (Hands-on) | □ Mentoring |
| □ Project work / research | □ Others *(please specify)* : ______________________________________ | |

**(D) PREFERRED LEARNING METHODS IN ANTIMICROBIAL STEWARDSHIP**

*Kindly place a* ***TICK (✓)*** *at* *your preferred learning methods based on the options provided, or you may specify other options which are not listed here. You are allowed to choose more than 1 option for this question.*

| □ CME / seminar / workshop /  course / conference | □ Self-learning / reading | □ Journal clubs / study groups |
| --- | --- | --- |
| □ Peer discussions | □ Experiential learning  (Hands-on) | □ Mentoring |
| □ Project work / research | □ Others *(please specify)* : ______________________________________ | |

**(E) BARRIERS TO LEARNING IN ANTIMICROBIAL STEWARDSHIP**

*Kindly place a* ***TICK (✓)*** *at your current barriers to learning in antimicrobial stewardship based on the options provided, or you may specify other options which are not listed here. You are allowed to choose more than 1 option for this question.*

| □ Lack of organizational support | □ Lack of funding |
| --- | --- |
| □ Unsure of the quality of available training | □ Lack of appropriate training |
| □ Lack of motivation | □ Lack of time to attend training |
| □ Others *(please specify)* :  _______________________________________________________________ | |

**- END OF QUESTIONNAIRE -**

**THANK YOU FOR YOUR PARTICIPATION !**
