# Supplementary material for: Self-perceived competence and training needs analysis on antimicrobial stewardship among government ward pharmacists in Malaysia
Source: JAC Antimicrob Resist. 2020 Jul 16;2(3):dlaa035. doi: 10.1093/jacamr/dlaa035 (PMC8210314; doi:10.1093/jacamr/dlaa035)
Supplement: dlaa035_Supplementary_Data [file dlaa035_supplementary_data.zip › Reviewer report 2.docx]

**Reviewer report 2 (R0 – review of original submission)**

**PLEASE NOTE: JAC-AMR aims to publish your anonymous peer review comments with the final article for transparency. Please indicate in the box below whether you give your consent for us to publish your peer review comments for this article.**

Yes

**Synopsis**

**1. If the article contains original data, does the synopsis adequately reflect the manuscript as a whole, with the most important aspects described (of the introduction, methods and key results, and a balanced conclusion)? If NO, what is required to improve the synopsis? Please use the box below.**

Yes

The synopsis reflects the manuscript and key results are
discussed. However, can the authors re-check the
calculation for response rate - specified in the results
section of the abstract, please? A total of 553 responses
out of 776 eligible government ward pharmacists would
give a response rate of 71.3%?

**Introduction**

**2. Does the introduction provide the necessary background information for readers to understand the article? (e.g. does it describe the problem, current state of knowledge, etc.) If NO, what is required to improve it? Please use the box below**

Yes

**3. Is the research hypothesis and/or aims/objectives of the study clearly stated in the introduction? If NO, what is required to improve it? Please use the box below**

Yes

**4. Is there superfluous/unnecessary text in the introduction? (Only text that is required for readers to understand why the study has been done should be included). If YES, what text is superfluous and should be removed? Please use the box below**

No

Considering the answers above and any other comments/advice you may have, please indicate what is required to improve the Introduction:

This paper assessed the self-perceived
competence and training needs on antimicrobial
stewardship (AMS) for government ward pharmacists in
Malaysia. It makes a significant contribution to this very
important field and highlights the learning needs
that can be addressed to better equip pharmacists to
improve AMS-related service.
The introduction provides the necessary
background. However, the authors can make some
minor sentence/grammar amendment to enhance flow in the introduction? Suggestions are made in the provided space under GENERAL.

**Methods**

**5. Were the methods used suitably robust and appropriate for the research question(s) and/or the aims/objectives of the study? If NO, the article should be rejected, but please advise the authors accordingly in the free-text box below and complete the review in full**

Yes

**6. Are the methods described in enough detail so the reader can understand what was done and interpret the results and conclusions appropriately?**

Yes

**7. Are the methods described in language that a reader who does not specialize in the area of the study/methods used will understand? For example, are the statistical approaches used described in language that non-statistician healthcare professionals will understand? If NO, please indicate what is required to improve the language in the methods section in the box below**

Yes

Considering the above and any other comments/advice you may have, please indicate other aspects of the methods section that could be improved in the free-text box below)

The study method is appropriate and described in detail
for understanding. The authors chose a Likert scale to
assess respondent's perceptions, which offers an
advantage of more levels of (dis)agreement compared
to a binary question model. Conducting a pilot with at
least 10% of the estimated sample size was a good
way of evaluating the research survey questionnaire/data collection tool before its actual use. Can the authors also offer a few more clarifications?

What changes were made to the data collection tool from results or findings of the pilot study?

On the value of Cronbach's alpha: if the items in a test are correlated to each other, the value of alpha is increased. The same is also obtainable if the test is long enough. What was obtainable with  this study - higher level of correlation, longer test, or both?

**Results**

**8. Given the methods used, are results described accurately, in enough detail and in an appropriate format to adequately inform the discussion section and the authors/readers conclusions? If NO, please indicate what is required to improve the results section in the box below**

Yes

**9. Based on what is described in the methods section, are data/results (that are likely to be available to the authors) missing from the current draft that you think should be presented to improve the manuscript?**

Yes

**10. Are Tables/Figures clear, required and do they add/complement the results text (they should not simply repeat what is in the text or vice versa)? If NO, please indicate what is required to improve the Tables/Figures in the box below**

Yes

Considering the above and any other comments/advice you may have, please indicate what is required to improve the results section:

This was mentioned in the comment on the synopsis but
also applies to the Results section. Can the authors recheck the calculation for response rate - specified in the results section of the abstract, please? A total of 553
responses out of 776 eligible government ward
pharmacists would give a response rate of 71.3%?

On point #9, is there room to present the additional table(s) (on preferred learning methods and barriers to learning in AMS), perhaps as supplementary material?


This following relates to both the RESULTS and DISCUSSION sections:

What are the ranks of the ward pharmacists who participated in the survey? Perhaps this can shed more light on the result (lines 241 to 243) and provide more information for discussion, with respect to lines 311-317? It may be that the pharmacists who have been ward pharmacists for longer may be in more advanced hierarchical positions, and therefore be involved in audit and evaluation?, but this may not necessarily be so?


With respect to the multitasking role of ward pharmacists (line 295), what other roles are ward pharmacists involved in? Do the authors know the proportion of time allocated to these other roles and the proportion of time allocated to ward pharmaceutical services in the study sites? This may perhaps be useful in the design/selection of training methods and appropriate AMS learning initiatives targeted at ward pharmacists.

**Discussion**

**11. Are the discussion and any conclusion(s) reasonable given the stated hypothesis/aims/objectives and results? If NO, please indicate how to improve the discussion in the box below**

Yes

**12. Is there superfluous/unnecessary text in the discussion? (Only text that directly relates to the results and understanding thereof in the context of existing published literature and the limitations of the study should be included.) If YES, please indicate what should be removed to improve the discussion in the box below**

No

**13. Is the Limitations sub-section adequate and reflective of the actual limitations of the study? ? If NO, please indicate how to improve the Limitations section in the box below**

Yes

Considering the above and any other comments/advice you may have, please indicate how to improve the discussion section:

The discussion supports and follows the objectives and methods of the study. It is also well presented to guide the  reader and his/her understanding of the work done. Below are comments for the authors' consideration:


The following relates to both the RESULTS and DISCUSSION sections:

With respect to the multitasking role of ward pharmacists (line 295), what other roles are ward pharmacists involved in? Do the authors know the proportion of time allocated to these other roles and the proportion of time allocated to ward pharmaceutical services in the study sites? This may perhaps be useful in the design/selection of training methods and appropriate AMS learning initiatives targeted at ward pharmacists.

What are the ranks of the ward pharmacists who participated in the survey? Perhaps this can shed more light on the result (lines 241 to 243) and provide more information for discussion, with respect to lines 311-317? It may be that the pharmacists who have been ward pharmacists for longer may be in more advanced hierarchical positions, and therefore be involved in audit and evaluation?, but this may not necessarily be so?


On the DISCUSSION text:
The discussion text is not superfluous but may benefit from some amendment. Suggestions for minor review on sentence/grammar are presented under GENERAL.

**General**

**14. Is referencing throughout the manuscript satisfactory? (for instance are there statements for which you believe a supporting reference should be cited?) If NO, please indicate how to improve referencing in the box below**

No

**15. Is the language used (i.e. spelling, grammar, etc.) of high enough quality to be published as is? If NO, please indicate how to improve the language within the article in the box below**

No

**16. Are ethics/other approvals appropriate/adequate and satisfactorily described? ? If NO, please indicate how to improve this in the box below**

Yes

Considering the above and any other comments/advice you may have, please advise the authors how to improve these aspects of the article.

This study addresses a very important topic, researched by robust methods and logically presented. The language may benefit from some amendment to improve quality and flow; please see suggestions below:


ABSTRACT:
Line 52: Replace 'Objective' with 'Objectives'.

Line 56: Insert 'of' after 'survey'.

Line 56: Delete 'was conducted from May to July 2018, involving'.

Line 58: Insert 'was conducted from May to July 2018' after '(HCTM)'.

Line 59: Please confirm that response rate is correctly calculated.

Line 61: Insert ',' after 'therapy'.

Line 72: Replace 'necessity' with 'need'.


INTRODUCTION:
Line 79: Replace 'is accounted as one of the' with 'is a'.

Line 79: Replace 'threats' with 'threat'.

Line 79: Delete the second 'the' before 'global'.

Line 79: Insert a comma (,) after 'health'.

Line 80: Replace 'with an estimated mortality of' with 'estimated to account for'.

Line 80: Delete 'people worldwide' and insert 'deaths annually'.

Line 86: Replace 'providing' with 'promoting'.

Line 87: Replace 'regime, including dosing' with 'regimen, dose’.

Line 92: Replace first sentence with ‘Among the stakeholders in AMS, the pharmacist played and continues to play a key role’.

Lines 93-94: Replace ‘were seen spearheading’ with ‘spearheaded’.

Line 94: Replace ‘taking’ with ‘took’, delete ‘a’, and insert a comma (,) after ‘co-leader’.

Line 95: Replace ‘Centre of’ with ‘The Centers for’.

Line 96: Insert ‘to’ between ‘interventions’ and ‘include’.

Line 99: Delete ‘that are’.

Line 100: Replace ‘durations of treatment’ with ‘duration’ and ‘detecting’ with ‘detection of’.

Line 102: Replace ‘pharmacists in playing critical roles’ with ‘the critical role of pharmacists’.

Line 104: Replace ‘giving’ with ‘and provision of’.

Line 105: Insert ‘AMS-related’ between ‘on’ and ‘issues’.

Line 105: Delete ‘pertaining to AMS’.

Lines 105 to 109 to read thus: ‘The involvement of pharmacists in AMS has been shown to contribute to improved timeliness of administration for in-ward antimicrobial agents (8), shortened hospital stay (9), significant reduction in antibiotic consumption (10), and a lower rate of hospital mortality and multidrug resistance (11).

Line 110: Replace ‘the success of pharmacists in influencing the’ with ‘these successful’.

Line 110: Replace ‘is’ with ‘are’.

Line 111: Insert “the” between “to” and “pharmacist’s”.

Line 113: Delete ‘had’.

Line 115: Replace ‘is’ with ‘are’.

Line 115: Insert ‘the’ before ‘CDC’.

Line 116: Replace ‘This includes’ with ‘These include’.

Line 118: Insert ‘with’ between ‘collaboration’ and ‘and’.

Line 119: Replace ‘to the’ with ‘of’.

Line 119: Insert comma (,) after ‘interventions’.

Line 125: Include a comma (,) after ‘methods’.

Line 126: Include a comma (,) after ‘AMS’.


METHODS:
Line 131: Replace ‘and ended on’ with ‘to’.

Line 134: Replace ‘Convenient’ with ‘Convenience’.

Line 135: Please confirm referenced author’s surname.

Line 143: Please check font size of first 3-4 words.

Line 145: Insert “Royal Pharmaceutical Society’s” between ‘the’ and ‘Expert’.

Lines 146-147: Delete ‘by the Royal Pharmaceutical Society’.

Line 151: Replace ‘response’ with ‘responses’.

Line 158: Replace ‘on’ with ‘of’.

Line 159-163 to read thus: ‘The final section of the questionnaire dealt with barriers to learning in AMS. The options were adapted from another learning needs analysis of clinical pharmacists and included constraints related to employer’s support, funding, time, appropriate training on offer, motivation, as well as uncertainty regarding the quality of available training.

Lines 177-181: Sentence to read thus: ‘Upon receipt of approval, further phone or electronic communication facilitated identification of pharmacy department representatives, access to a directory of ward pharmacists and their corresponding email addresses, as well feedback on preferred mode of completing the questionnaire (i.e., either via the online platform or by use of hard copies).’

Line 181: Insert ‘The’ before “URL’.

Line 185: Insert ‘a’ between ‘with’ and ‘different’.

Line 185: Replace ‘numbers’ with ‘number’.

Line 195: Replace ‘with’ with ‘and’.

Line 199: Delete ‘both’.

Line 200: Include a comma (,) after ‘Health’.

Line 201: Insert ‘the’ between ‘and’ and ‘Research’.

Line 203: Delete the first ‘the’.


RESULTS:
Line 208: This comment was included for the abstract as well – can the authors check the calculation for response rate, specified in the results
sections of the abstract and the manuscript, please? A total of 553 responses
out of 776 eligible government ward pharmacists would 3give a response rate of 71.3%?

Line 216: Replace ‘characteristic’ with ‘characteristics’ and ‘is’ with ‘are’.

Line 217: Replace ‘is’ with ‘was’.

Line 218: Replace ‘is’ with ‘was’.

Line 220: Replace “bachelor” with “bachelor’s”.

Line 241: Delete ‘the’ at the end of the line.

Line 242: Replace ‘was the’ with ‘were’.

Line 262: Replace ‘seminar, course or workshop was’ with ‘seminars, courses or workshops were’.

Line 263: Replace ‘method’ with ‘methods’.

Line 264: Replace ‘is’ with ‘was’.

Line 266: Delete ‘another’.

Line 267: Delete the comma (,) after ‘learning’.

Line 268: Replace comma (,) after ‘participation’ with a semi-colon (;).

Line 268: Delete ‘with’.

Line 268: Delete ‘with’ and replace ‘Around’ with ‘About’.

Line 271: Delete ‘top three most’.

Lines 273-274: Replace ‘CME session, seminar, course or workshop’ with ‘CME sessions, seminars, courses or workshops’.

Lines 275-276: Replace ‘remained the second and third most preferable learning method, respectively’ with ‘ranked second and third, respectively, in order of preference.

Line 277: Replace ‘was preferred among 52.3% (n=289) of the respondents’ with ‘ranked fourth (52.3%, n=289)’

Line 279: Delete full stop (.).

Line 279:  Delete ‘40.1% (n=222) of the respondents preferred’

Line 279: Insert ‘and’ before ‘journal’.

Line 280: Insert ‘(40.4%, n=222)’ after ‘groups’.

Line 281: Insert a full stop (.) after the first bracket and delete the comma(,).

Line 281: Replace ‘and there were another five’ with ‘Five’.

Line 281: Delete ‘who’.

Lines 286-294 to read thus: ‘A few barriers to learning in AMS were identified in this study. More than half of the respondents acknowledged the lack of appropriate training as a major hindrance to learning in AMS. Lack of time to attend training (n=264, 44.5%) and lack of funding (n=235, 42.5%) were the second and third highest ranked factors, respectively. About 35.3% of the respondents (n=195) attributed barriers in learning to lack of organizational support while 21.9% (n=121) expressed uncertainty regarding the quality of available training. The least acknowledged factor was lack of motivation to learn about AMS which was recorded from 14.1% of the respondents (n=78).’

Line 295: Replace ‘multitasking’ with ‘the multitasking role’.


DISCUSSION:

The text is not superfluous, but may need some minor amendment, suggested below:

Lines 305-308: Please reword the sentence for easier and better comprehension.

Line 308: Replace 'in this study' with 'of this study'.

Lines 311-317 to read thus: ‘The relatively lower level of ward pharmacists’ involvement in clinical audit and evaluations related to antimicrobial pharmacy service suggests that such activities were rarely performed in the respective facilities, or that they might be assigned to ward pharmacists in more advanced hierarchical positions. As audit and evaluation is a key element of AMS, with the potential to address knowledge gaps among ward pharmacists, the findings of this study emphasize the need to integrate audit and evaluation in ward pharmacists’ roles.

Line 320: Replace 'suggested' with 'suggests'.

Line 325: Replace 'provides higher chances' with 'provides more opportunities'.

Line 331: Replace 'on' with 'in'.

Line 333: Delete 'current'.

Line 334: Replace 'with' with 'to'.

Line: 336: Replace 'a' with 'the'.

Line 338: Replace 'inputs' with 'input'.

Line 340: Replace 'find' with 'found'.

Line 341: Delete 'as'.

Line 342-343: 'Replace 'tendency of underestimating' with 'tendency to underestimate'.

Line 343: Replace 'tend to score' with 'scored'.

Line 344: Replace 'assessments' with 'assessment'.

Line 345: Delete 'Findings in'. Replace 'popularity' with 'preference'.

Lines 354-355: Replace "a few reasons, mainly due to pharmacists being reluctant in participating research activities" with "pharmacists' reluctance to participate in research activities".

Line 356: Replace ‘many’ at the beginning of the line with ‘two recent’ and add an apostrophe (') after 'pharmacists'.

Line 361 Replace ‘There was’ with ‘A’.

Line 361 to 363: Table for this - perhaps can be added to supplementary material(s)?

Line 377: Replace ‘of’ with ‘to’.

Line 392: Replace 'another two' with 'two other'.

Line 398: Replace ‘answering’ with ‘completing’.


CONCLUSION:
Line 407: Replace the second ‘in’ with ‘of’.

Line 410: Replace ‘competent’ with ‘competence’.

Lines 410-412: Rewrite the sentence to read this: ‘The preferred AMS learning methods were CME, seminars, courses or workshops; however, access to these are hampered by the lack of time and funding as well as a paucity of appropriate training available.

Line 412: Replace ‘strongly suggest’ with ‘highlight’.

Line 413: Replace ‘necessity’ with ‘need’.


REFERENCES
Please see some comments on the references below:

Line 431: Please check ref. # 3: would the reference below (formatted according to the author's or journal's referencing style, of course) be more suitable?

Society for Healthcare Epidemiology of America, Infectious Diseases Society of America, & Pediatric Infectious Diseases Society. (2012). Policy Statement on Antimicrobial Stewardship by the Society for Healthcare Epidemiology of America (SHEA), the Infectious Diseases Society of America (IDSA), and the Pediatric Infectious Diseases Society (PIDS). Infection Control and Hospital Epidemiology, 33(4), 322-327. doi:10.1086/665010

Line 438: Please complete the article title for reference #4: "stewardship programs" missing from the title?

Line 475: Please check ref # 14 again for the author’s correct surname?

Line 518: Reference #27 - include the additional authors of the publication and complete the title of the publication (the word 'pharmacist' missing from the title?).

**Opinion and review articles only**

**17. Please advise the authors in the box below on how to improve their opinion or review article:**

|  **Recommendation** | |
| --- | --- |
|  | Accept (as is with no revisions) |
|  | Minor revision (with anticipation of subsequent acceptance if revisions are adequate) |
|  | Major Revision |
|  | Reject |

| **Would you be willing to review a revision of this manuscript?** | |
| --- | --- |
|  | Yes |
|  | No |

**Comments**

 Comments to the Author

If you have any additional comments to the authors about any aspect of the manuscript not covered in the structured review above, please place these in the box below. If you have no additional comments, please say “No additional comments” in box.

Can the authors also check wording in tables, please? Do the following words in the tables need to be reviewed?
- "Bachelor" (Table 1, page 22 of 26),
- "pharmacodynamics", "Advises" and "pharmacoeconomics" (Table 2, page 24 of 26).
